# Applied Molecular-Based Quality Control of Biobanked Samples for Multi-Omics Approach

**DOI:** 10.3390/cancers15143742

**Published:** 2023-07-24

**Authors:** Anna Michalska-Falkowska, Jacek Niklinski, Hartmut Juhl, Anetta Sulewska, Joanna Kisluk, Radoslaw Charkiewicz, Michal Ciborowski, Rodryg Ramlau, Robert Gryczka, Cezary Piwkowski, Miroslaw Kozlowski, Borys Miskiewicz, Przemyslaw Biecek, Karolina Wnorowska, Zofia Dzieciol-Anikiej, Karine Sargsyan, Wojciech Naumnik, Robert Mroz, Joanna Reszec-Gielazyn

**Affiliations:** 1 Biobank, Medical University of Bialystok, 15-269 Bialystok, Poland; zofia.dzieciol@umb.edu.pl (Z.D.-A.); joasia@umb.edu.pl (J.R.-G.); 2 Indivumed Services, 20251 Hamburg, Germany; juhl.hartmut@indivumed.com; 3 Department of Clinical Molecular Biology, Medical University of Bialystok, 15-269 Bialystok, Poland; jacek.niklinski@umb.edu.pl (J.N.); joanna.kisluk@umb.edu.pl (J.K.); radoslaw.charkiewicz@umb.edu.pl (R.C.); 4 Center of Experimental Medicine, Medical University of Bialystok, 15-369 Bialystok, Poland; 5 Clinical Research Centre, Medical University of Bialystok, 15-276 Bialystok, Poland; michal.ciborowski@umb.edu.pl; 6 Department of Oncology, Poznan University of Medical Sciences, 60-569 Poznan, Poland; rodrygramlau@ump.edu.pl (R.R.); robert-gryczka@wp.pl (R.G.); 7 Department of Thoracic Surgery, Poznan University of Medical Sciences, 60-569 Poznan, Poland; cpiwkow@ump.edu.pl; 8 Department of Thoracic Surgery, Medical University of Bialystok, 15-269 Bialystok, Poland; miroslaw.kozlowski@umb.edu.pl (M.K.); borys.misiewicz@umb.edu.pl (B.M.); 9 Faculty of Mathematics and Information Science, Warsaw University of Technology, 00-662 Warsaw, Poland; przemyslaw.biecek@pw.edu.pl; 10 Department of Medical Pathomorphology, Medical University of Bialystok, 15-269 Bialystok, Poland; karolina.wnorowska@umb.edu.pl; 11 Department of Paediatrics and Pediatric Neurology, Jedrzej Sniadecki Independent Public Healthcare Centre Regional Hospital, 15-278 Bialystok, Poland; 12 Department of Rehabilitation, Medical University of Bialystok, 15-089 Bialystok, Poland; 13 International Biobanking and Education, Medical University of Graz, 8036 Graz, Austria; karine.sargsyan@medunigraz.at; 14 Cancer Biobank at Cedars-Sinai Medical Center, Los Angeles, CA 90048, USA; 15 1st Department of Lung Diseases and Tuberculosis, Medical University of Bialystok, 15-540 Bialystok, Poland; wojciech.naumnik@umb.edu.pl; 16 2nd Department of Lung Diseases and Tuberculosis, Medical University of Bialystok, 15-540 Bialystok, Poland; robert.mroz@umb.edu.pl

**Keywords:** quality management, multi-omics research, translational research, best practices, omics-grade biospecimens

## Abstract

**Simple Summary:**

This study highlights the significance of quality assurance in biobanking facilities, specifically in the context of high-throughput research and novel molecular techniques. We established specific quality management workflows utilizing biospecimens collected from oncological patients in Polish clinics. The process for serum/plasma samples involved essential steps, such as monitoring hemolysis, controlling RNA extraction, assessing cDNA library quality and quantity, and evaluating NGS raw data before bioinformatics analysis. Pathologic evaluation of fresh frozen tissue samples was conducted to verify histological images and determine tumor content. Consequently, we developed workflows for preanalytical quality control of serum/plasma and fresh-frozen tissue samples, which can be tailored to individual biobank capabilities. These integrated molecular-based quality control methods ensure that biobanking procedures fulfill the requirements of high-throughput assays, offering access to biospecimens of confirmed omics-grade quality. We strongly recommend implementing these workflows for specimen qualification before conducting multi-omics assays, as they exhibit exceptional sensitivity and specificity.

**Abstract:**

Biobanks are vital for high-throughput translational research, but the rapid development of novel molecular techniques, especially in omics assays, poses challenges to traditional practices and recommendations. In our study, we used biospecimens from oncological patients in Polish clinics and collaborated with the Indivumed Group. For serum/plasma samples, we monitored hemolysis, controlled RNA extraction, assessed cDNA library quality and quantity, and verified NGS raw data. Tissue samples underwent pathologic evaluation to confirm histology and determine tumor content. Molecular quality control measures included evaluating the RNA integrity number, assessing cDNA library quality and quantity, and analyzing NGS raw data. Our study yielded the creation of distinct workflows for conducting preanalytical quality control of serum/plasma and fresh-frozen tissue samples. These workflows offer customization options to suit the capabilities of different biobanking entities. In order to ensure the appropriateness of biospecimens for advanced research applications, we introduced molecular-based quality control methods that align with the demands of high-throughput assays. The novelty of proposed workflows, rooted in innovative molecular techniques, lies in the integration of these QC methods into a comprehensive schema specifically designed for high-throughput research applications.

## 1. Introduction

The application of next-generation sequencing (NGS) has revolutionized our understanding of cancer development and progression. Identifying cancer-related mutations and resistance biomarkers improved our knowledge of the molecular basis of different cancer types [1]. Although genomics gave the foundation of present personalized medicine in oncology, it is becoming increasingly clear that determining molecular phenotype is essential to our understanding of tumor biology and may lead us to the new era of precision medicine [2]. To personalize oncology, we must go beyond genetics and understand the complexity of cancer through multi-omics analysis. Protein posttranslational modifications give information about epigenetic inheritance and chromatin relaxation or compaction [2,3]. Metabolomics is also a crucial part of this puzzle since the provided results are the most dependent on the environment and lifestyle. Therefore, it gives information about genotype–phenotype and genotype–environment interactions [4]. Consequently, applying all omics technologies to study particular types of cancer, together with applying advanced bioinformatics to examine connections between different components of systems biology, is crucial to reach the next level of precision oncology. To ensure valid, accurate, and reproducible results of biomedical studies, we need to keep the standardized collection, processing, storage, and shipment of biospecimens—and biobanks are the answer to this necessity. The idea of personalized medicine in cancer treatment was the trigger for accelerating the progress of biorepositories organization through the vital need for high-quality biospecimens. Development of biobanks entailed improving technological hinterland for efficient management of large amounts of samples, clinical data, and big data from multi-omics assays. The usefulness and reliability of molecular data obtained from the studies using biospecimens depend on the quality of starting material. Preanalytical factors, such as ischemia time, type and time of fixation, storage temperature, and length of storage, are only a few variables that can result in poor quality and low yields of DNA, RNA, and protein, affecting gene and protein expressions and, consequently, metabolites level. As epigenomics, metabolomics, and proteomics were recognized as the backbone of cancer research, biobanks became fundamental units involved in biomarker discovery [5]. Therefore, the establishment of the Biobank at the Medical University of Bialystok, Poland (MUB Biobank) arose from the need to provide high-quality samples for multi-omics investigations. This solution aimed to integrate biospecimens collected from oncological patients with comprehensive interviews, clinical data, and omics data. Such a complex project required support from experienced partners to implement Biobanking Best Practices efficiently, and it was achieved through cooperation with Indivumed. There are numerous freely available resources with best practices and guidelines for biobanking, covering areas such as collection, processing, storage, and quality control of biospecimens, aimed at ensuring the quality, integrity, and ethical use of biological specimens [6,7,8]. As the integration of multi-omics approaches within biobanking is an emerging field, and while specific recommendations are still evolving, we have identified the demand for precise and affordable molecular-based quality control methods (QC) for liquid and tissue samples. The workflows presented in this study offer novel approaches for QC in high-throughput research by incorporating visual hemolysis assessment, spectrophotometric analysis, and molecular-based models to ensure the utility of biospecimens for downstream analysis. It is important to highlight the lack of integrated guidelines specifically addressing the QC of biospecimens for high-throughput research. While individual QC methods may exist, the development of a holistic workflow that combines multiple techniques and provides detailed protocols is an innovative contribution. By addressing this gap, the presented workflows offer researchers a comprehensive and standardized approach to assess sample quality and enhance the reliability of high-throughput assays.

## 2. Materials and Methods

MUB Biobank’s organization was possible by founding the MOBIT project from National Centre for Research and Development in the framework of the STRATEGMED II Program. The MOBIT project aimed to combine biobanking with high-throughput methods to create a reference model for early lung cancer diagnosis and personalized treatment [9]. Within the cancer-oriented section of MUB Biobank, three units were established in Poland: Central Biobank in Bialystok and two Satellite Biobanks—one in Greater Poland Center of Pulmonology and Thoracic Surgery (Poznan University of Medical Sciences) and one in the Gynecology Clinic at District Specialist Hospital in Olsztyn. As widely described, we concluded that integrated cooperation between biobanks makes establishing standardized collections the essential resource for multi-omics research aimed at developing personalized cancer treatments [5,10]. It is important to emphasize that teams from Satellite Biobanks used the same laboratory equipment as in the Central Biobank to minimize the risk of technical bias that could lead to invalid results in large-scale omics research [11].

To maintain the comparability and reproducibility of research conducted on samples stored in a biorepository, standard operating procedures (SOPs) were prepared to regulate every step of biobanking. Moreover, QC forms are used for recording sample-connected processes. Both SOPs and QC forms are subjected to periodic review to maintain compliance with the latest scientific reports.

Documenting the exact time of resection start, vessel ligation, resection end, and tissue sample preservation is essential for monitoring warm and cold ischemia. The ideal sample represents in vivo conditions, so efforts are made to shorten the period between tissue resection and preservation. Recently published reports describe the impact of postoperative ischemia duration on tissue sample biology, e.g., gene expression and RNA stability [12,13]. In MUB Biobank, tissue sampling is performed just after tumor resection by a pathologist or qualified study nurse present in the operating room. The samples from the tumor margin, tumor center, and normal tissue (not a case in brain tumors) are cut by the pathologist in pursuance of the scheme: one sample is to be stored in liquid nitrogen and another sample is to be put in buffered formalin; one fresh-frozen sample has a mirror sample in the form of formalin-fixed paraffin-embedded (FFPE) tissue. The samples are placed in the barcode-labeled cryotubes and tubes with formalin, according to their designation. Tissue samples in cryotubes are immediately placed into the vapor phase of liquid nitrogen for on-site snap-freezing and transferred to MUB Biobank cryotanks for final storage. The mirror samples preserved in buffered formalin are then processed into FFPE blocks in the Pathology Department. Tissue samples are collected and secured in liquid nitrogen and formalin prior to the diagnostic process to possibly shorten the cold ischemia time.

**Figure 1:**
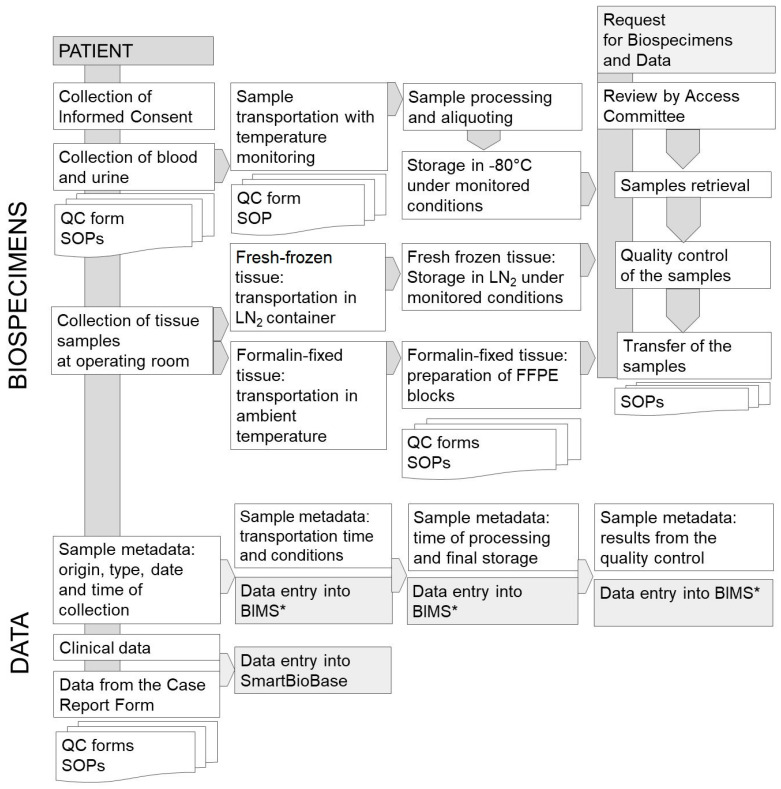
The process of biospecimens and data collection in the oncology section of MUB Biobank. * BIMS—Biobank Information Management System.

To compile large amounts of data in a user-friendly environment, it is necessary to digitalize the paper reports in a database. During the MOBIT Project, the SmartBioBase—a platform for collecting, integrating, and analyzing clinical and omics data—was created [14]. This environment is a digital storage space with data processing and data mining applications. The integrated process of biospecimens and data collection is presented in Figure 1.

**Figure 2:**
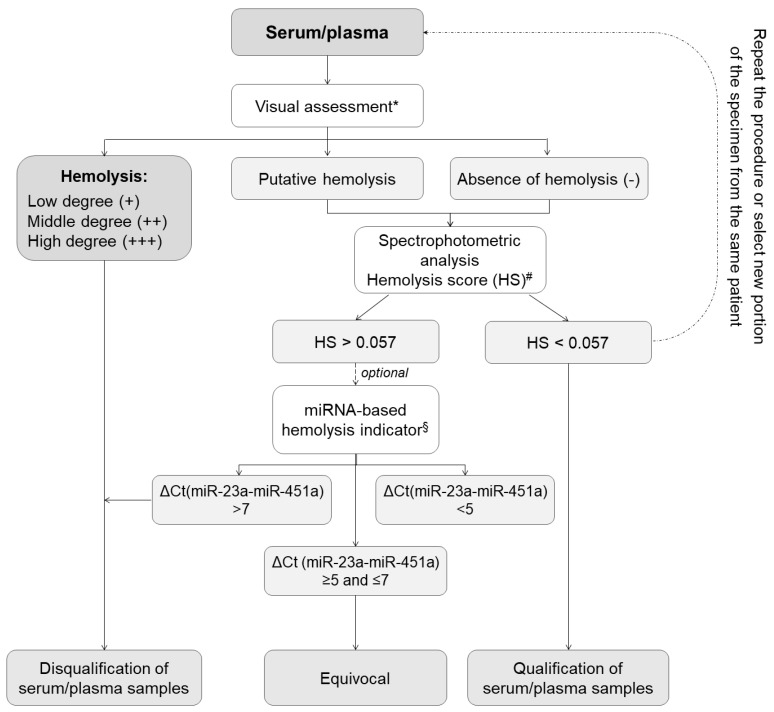
Scheme of quality control process based on analytical techniques for monitoring hemolysis in serum/plasma samples. * The visual assessment of potential blood hemolysis (rupturing of erythrocytes) was performed according to the following scale: −, no discernable hemolysis (serum/plasma color is pale yellow or yellow)—sample suitable for further processing; +/−, suspected hemolysis (serum/plasma color is pale pink)—sample may be suitable for further processing but requires additional confirmation by spectrophotometric technique and optionally qPCR-based approach. +/++/+++, low, medium, or high degree of hemolysis (serum/plasma tinted red in varying degrees)—sample is not suitable for further processing; refrain from carrying out further procedure. # Spectrophotometric analysis based on oxyhemoglobin absorbance measurements at λ = 414 nm and at λ = 385 nm (as a lipemia indicator) using NanoDrop 2000c spectrophotometer with cuvette module (100 µL) (Thermo Fisher Scientific, Waltham, MA, USA) [15]. Range: Hemolysis score (HS): HS < 0.057—non-hemolyzed plasma/serum specimens; HS > 0.057 hemolyzed plasma/serum samples. § Quantification RT-PCR method based on hsa-miR-451a and hsa-miR-23a-3p expression levels [16]. miRNA-based hemolysis indicator formula: ΔCt (miR-23a–miR-451). Range: ΔCt > 5—possible erythrocyte miRNA contamination; ΔCt > 7–8 or more—high risk of blood hemolysis.

Apart from detailed sample collection, processing, and storage documentation, measuring and analyzing key process indicators plays an essential role in quality improvement. Accordingly, we developed molecular-based models for QC of liquid and tissue biospecimens to ensure their utility for high-throughput research. As shown in Figure 2, the first step of the proposed QC schema of serum and plasma is the visual hemolysis assessment performed according to the three-point scale that indicates an absence of hemolysis, suspected hemolysis, or hemolysis at different degrees. The second quick, and low-cost technique to monitor hemolysis in serum/plasma is the spectrophotometric analysis based on oxyhemoglobin absorbance measurements at λ = 414 nm and λ = 385 nm [15]. Distinct values of hemolysis score (HS) indicate the presence of free hemoglobin and can be used to disqualify samples affected by hemolysis before time-consuming and expensive processing [16]. The validation of hemolysis analysis involves several key steps, including sample selection, visual assessment, spectrophotometric analysis, and quantification RT-PCR method. We collected a diverse set of samples including 347 serum and 694 plasma aliquots stored in MUB Biobank, ranging from non-hemolyzed to severely hemolyzed samples. These samples were carefully selected to represent the full spectrum of hemolysis and enable robust validation of the spectrophotometric hemolysis analysis.

The samples were visually assessed by expert technicians using standard hemolysis assessment protocol as presented in Figure 2. The samples with a volume of 2 μL each were then subjected to hemolysis analysis using the NanoDrop 2000c (Thermo Fisher Scientific, Waltham, MA, USA) instrument, which measured the absorbance at λ = 414 nm and λ = 385 nm wavelengths to determine the hemolysis score. The obtained hemolysis scores were recorded for each sample. According to the literature describing spectrophotometrically based systems for hemolysis analysis [15], lipemia-independent HS less than 0.57 indicates a low level of hemolysis in the sample and suggests minimal to no release of hemoglobin from red blood cells. An HS greater than 0.57 indicates that there is a significant release of hemoglobin from lysed red blood cells, potentially affecting the quality and integrity of the sample. Samples with higher hemolysis scores may require additional consideration or exclusion from certain analyses to ensure accurate and reliable results. For the further analysis of hemolysis in samples with HS greater than 0.57, we used the quantification RT-PCR method based on hsa-miR-451a and hsa-miR-23a-3p expression levels. Hsa-miR-451a is abundant in red blood cells, while hsa-miR-23a-3p is less affected by hemolysis. Therefore, the ratio of hsa-miR-451a to hsa-miR-23a-3p expression levels can serve as an indicator of the degree of hemolysis in the sample. The plasma/serum samples with miR-451/miR-23a ratio higher than 7 were recognized as hemolytic, while samples with miR-451/miR-23a ratio lower than 5 were described as non-hemolytic. The miR-451/miR-23a ratio between 5 and 7 indicated a moderate level of hemolysis in the sample, reflecting some degree of red blood cell lysis and release of miR-451 into the sample [16].

Analytical methods for assessing blood hemolysis are necessary to provide high-quality serum/plasma samples for molecular assays because even small amounts of cellular contamination in these biospecimens can affect the miRNA expression profile. Moreover, increased concentration of free hemoglobin and other molecules (e.g., bilirubin) released from red blood cells may affect proteomics and metabolomics profiles. While these methods primarily address the specific issue of hemolysis, they are integral components of a broader QC framework for omics approaches to ensure the reliability and accuracy of omics data, enhance data reproducibility, and facilitate valid comparisons across different studies.

The first step of the QC process developed for fresh-frozen tissue includes the histological examination of biospecimens. All samples were subjected to frozen section histology for precise tissue sectioning. Cut specimens were mounted on glass slides and stained with hematoxylin and eosin to allow microscopic examination of sample morphology, including the pathological interpretation of the image and calculation of tumor content. For the inclusion criteria for further molecular assays, ≥50% was determined as sufficient tumor content in the tissue samples collected from the tumor center and the tumor periphery [17]. Adequate tumor DNA content is essential for the accurate interpretation of molecular test results from the analyzed sample. Insufficient tumor DNA proportions can lead to missed detection of genetic abnormalities since the signal from aberrant DNA in tumor cells may be overshadowed by an excess of normal DNA from non-tumor cells. The recommended percentage of tumor content for omics studies can vary depending on several factors, including the specific research question, the type of omics analysis being conducted, and the tumor type under investigation. While there is no universally agreed-upon threshold, a common guideline is to aim for a tumor content of at least 20–30% or higher [18].

**Figure 3:**
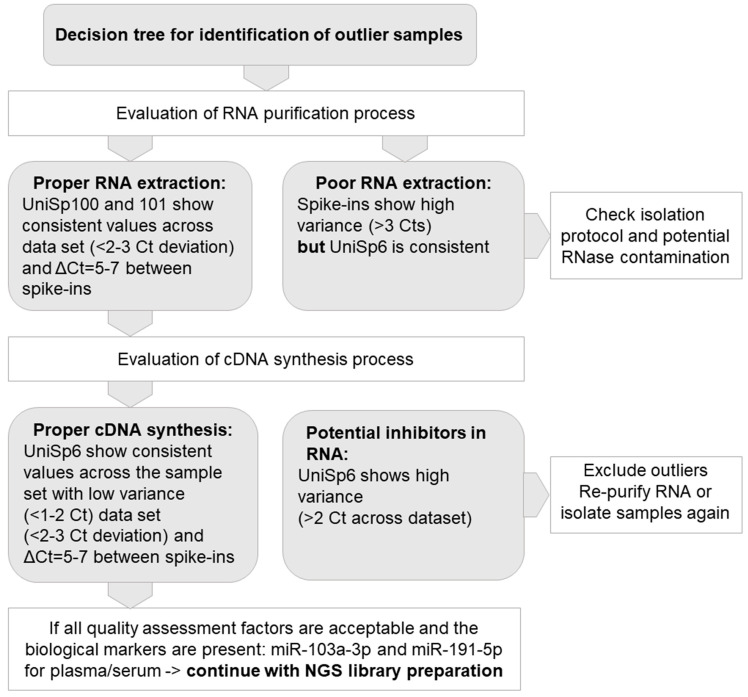
Model of quality control (QC) for RNA extracted from plasma/serum samples.

The assessment of RNA integrity is a critical step in obtaining meaningful gene expression data. The RNA integrity number (RIN) scale ranges from 1 (degraded) to 10 (intact) and is a widely used metric to assess the quality and integrity of RNA samples for omics studies, particularly transcriptomics. While there is no universally agreed-upon threshold for the RIN value, a common recommendation is to aim for a RIN value of 7 or higher [19]. During the selection of biospecimens from MUB Biobank, RNA isolates with RIN ≥ 7 are used for omics analysis. Key QC parameters of RNA purified from the serum samples include the efficiency of RNA extraction, absence of nucleases, and any inhibitors of enzymatic reactions. To reliably assess RNA quality from serum and plasma samples, the qPCR method based on a set of synthetic RNA spike-ins and endogenous microRNA assays were used. All samples were controlled using the ExiSEQ-NGS sample QC kit–small RNA/microRNA (Exiqon Inc. Vedbaek, Denmark) before proceeding with microRNA profiling by NGS (Figure 3).

QC of RNA extracted from fresh-frozen tissue samples from tumor periphery, tumor center, and normal adjacent tissue included the isolation of total RNA from fresh-frozen tumor samples using mirVana miRNA Isolation Kit (Thermo Fisher Scientific, Waltham, MA, USA). To monitor the comparability and reproducibility of the whole process from RNA isolation to sequencing, a mix of 5′ phosphorylated microRNAs of different concentrations (ExiSEQ-NGS Spike-ins, Exiqon Inc. Vedbaek, Denmark) was added to each RNA after the extraction of nucleic acids process. RNA quantity and quality were assessed using a UV/VIS spectrophotometer NanoDrop 2000c (Thermo Fisher Scientific, Waltham, MA, USA). The level of integrity required for the NGS analysis (RIN ≥ 7) was determined for the extracted RNA using Agilent RNA 6000 Nano Kit on Bioanalyzer 2100. Libraries for NGS were prepared using NEXTflex^®^ Small RNA-Seq Kit v3 (PerkinElmer, Waltham, MA, USA). The complementary DNA (cDNA) libraries were assessed qualitatively using a high-sensitivity DNA Chip assay on Bioanalyzer 2100. Size selection of the libraries to obtain the appropriate cDNA products (~150 base pairs) for NGS was performed on apparatus Blue Pippin (SageScience Inc., Beverly, MA, USA). Then, quantities of cDNA libraries were assessed using a KAPA Library Quantification Kit for Illumina Platforms (Roche, Basel, Switzerland). The NGS of the prepared samples was carried out on HiSeq 4000 Illumina. QC of sequencing data was performed using the mixture of spike-ins, added to the samples after total RNA isolation. The set of ExiSEQ NGS spike-ins allowed for thorough QC of the NGS data by assessing the reproducibility and linearity of the reads mapped to exogenous sequences.

**Figure 4:**
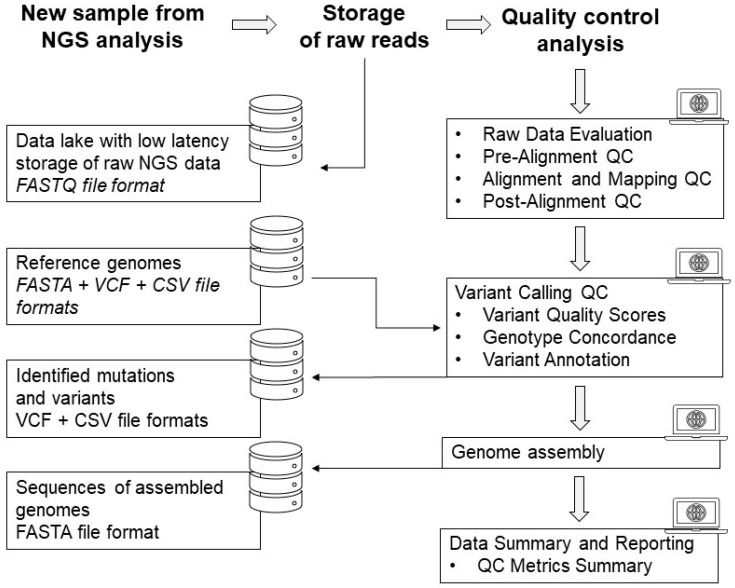
The process of raw data processing from the NGS analyses to final genome assembly and data reporting.

Continuous evaluation and improvement of QC protocols are necessary to adapt to evolving technologies and best practices in NGS. It ensures that the data used for downstream analyses is of sufficient quality to produce more accurate and meaningful interpretations, reducing the risk of false discoveries or incorrect conclusions. The QC process for NGS starts with the storage of raw FASTQ data in a dedicated database within the Bioinformatic Cluster managed by MUB. Data quality checks are performed on the reads to assess their overall quality, including metrics such as sequencing depth, read length, and base quality scores. The next step involves mapping the reads to a reference genome to determine their alignment and identify any potential variations or mutations. This is followed by variant calling where specific genetic variations, such as single nucleotide polymorphisms (SNPs) or insertions/deletions (indels), are identified and annotated. Additionally, genome assembly is performed to reconstruct the full genome sequence based on the mapped reads. This step allows for the identification of structural variations and complex genomic rearrangements. Figure 4 illustrates the entire process of processing NGS data, starting from the raw NGS data and progressing through data quality checks, read mapping, variant calling, and genome assembly. This visual representation provides a clear understanding of how the NGS data is processed to identify the mutational status. By validating this QC process, including regular evaluation and improvement, we ensure that the NGS data generated is of high quality and can be confidently used for downstream analyses and interpretations.

## 3. Results

Biospecimen and data distribution for the multi-omics approach is an integral part of biobank sustainability and participation in scientific projects. Before the preparation of molecular analysis, the repository provides necessary information about the specimens, including sample quality reports.

For the implementation of Best Practices in the QC of serum, plasma, and tissue samples prior to their application in multi-omics assays, we developed comprehensive workflows. As results of high-throughput studies strongly depend on the quality of the biospecimens, the essential part of plasma and serum QC should include an assessment of hemolysis, as described above in the Materials and Methods section. Following RNA extraction from serum and plasma, we included the molecular assessment of RNA in the QC workflow. The efficiency and correctness of the RNA purification process were analyzed on the basis of deviations in cycle threshold (Ct) in the set of synthetic RNA spike-ins. To identify the outlier samples with quality not sufficient to perform further omics assays, we determined the set of quality assessment factors, as presented in Figure 3. The proposed workflow was developed and tested with the use of 347 serum and 694 plasma samples stored in MUB Biobank in the process of biospecimen qualification to genomics, transcriptomics, metabolomics, and proteomics assays. Hemolysis was observed in about 1.3% of the samples, and these samples were not forwarded for omics analyses. Based on the visual inspection suspected hemolysis was noted in 47% of the samples, but after oxyhemoglobin absorbance measurements, all these samples were allowed for omics assays.

In the process of developing the QC workflow for fresh-frozen tissue samples collected from the tumor center, tumor periphery, and normal adjacent tissue, the first step included histopathological evaluation of collected biospecimens. After meeting the inclusion criteria, RNA from tissue samples was isolated and subjected to QC that in the primary step included the assessment of RIN. For all tissue samples, the checkpoint of QC was performed by nucleic acids fluorometric quantification (Qubit, Thermo Fisher Scientific, Waltham, MA, USA). Detailed data on the average concentration values are presented in Table 1. RIN below 7 was detected in 0.93% of 432 tested samples. The next checkpoint was carried out by PreSeq QC analysis with a real-time PCR method to determine the quality of starting material and for predicting the success of NGS.

**Figure 5:**
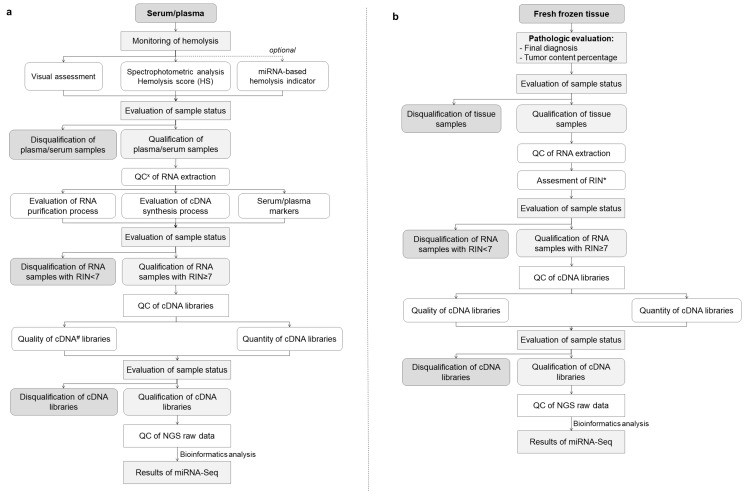
(**a**,**b**) The algorithm developed during the MOBIT project with an essential preanalytical and analytical QC process for optimal biobanking of serum/plasma samples (**a**) and tissue samples (**b**). x QC—quality control, # cDNA—complementary DNA, * RIN—RNA integrity number.

Samples with RIN ≥ 7 were then qualified for the next stages of evaluation, as described in the Materials and Methods section and presented in Figure 5b.

A total number of 218 fresh-frozen tissue samples from MUB Biobank were used in the process of QC workflow development, including 147 samples collected from the lung tumor periphery and 71 from the lung tumor center. The final diagnosis made on the basis of microscopic tissue examination was lung adenocarcinoma in 121 cases and squamous cell carcinoma in 97 cases. A total number of 152 fresh-frozen tissue samples collected from adjacent normal tissue were used as control samples.

The combined workflow developed to address essential preanalytical and analytical QC in miRNA research conducted on liquid and tissue samples is shown in Figure 5a,b.

**Figure 6:**
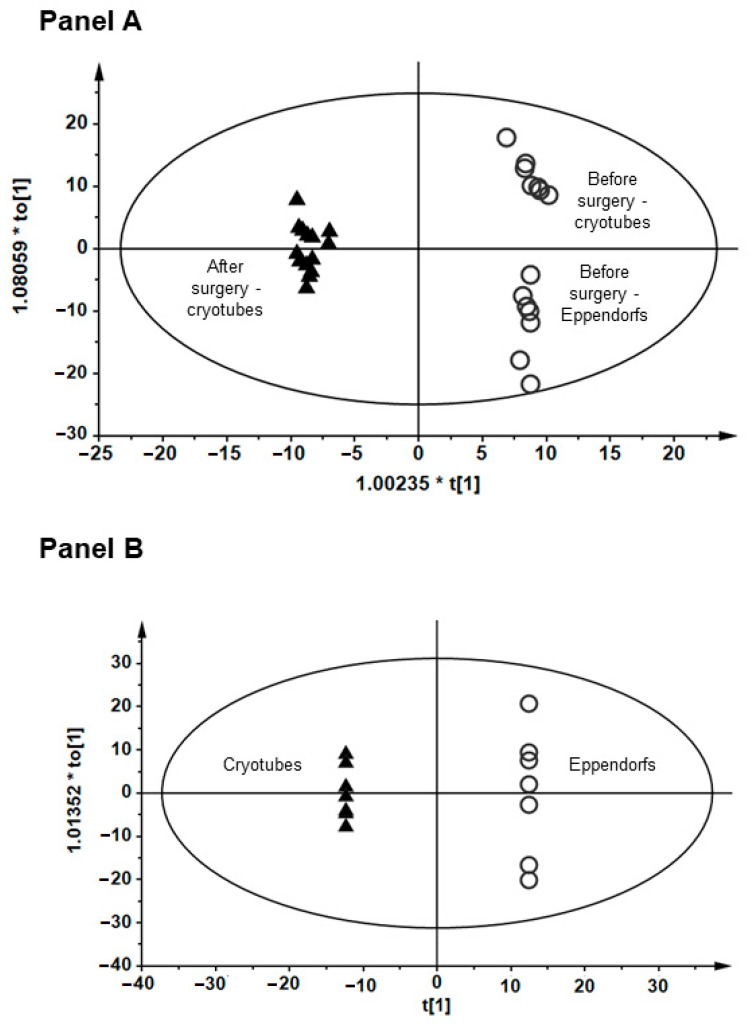
Orthogonal partial least squares discriminant analysis (OPLS-DA) models showing the classification of plasma metabolic fingerprints obtained with liquid chromatography quadrupole time-of-flight mass spectrometry (LC-QTOF-MS) method. (**Panel A**) (R2 = 0.897, Q2 = 0.364) shows the separation between plasma samples collected from patients before and after the tumor resection surgery. (**Panel B**) (R2 = 0.993, Q2 = 0.5364) shows the separation between samples stored in cryotubes and Eppendorf tubes.

To avoid the potential influence of preanalytical aspects on plasma/serum metabolome, plasma metabolic fingerprinting was applied with the use of samples collected from patients the day before lung cancer surgery and three months after this surgical procedure. For the purpose of this test, two sets of plasma samples were prepared: one with plasma stored in cryotubes and one with plasma stored in Eppendorf tubes. The results of plasma metabolic fingerprinting performed with liquid chromatography quadrupole time-of-flight mass spectrometry (LC-QTOF-MS) method revealed that samples collected from patients before surgical tumor resection were separated from the samples obtained from the same patients after the surgery (Figure 6 Panel A). There was also a clear separation of samples collected before surgery (Figure 6 Panels A and B) related to plastic material used to store plasma samples. Observed separation can be partly associated with individual variability. However, this variability was not that significant among the same patients after the surgery. Moreover, plasticizers and other substances may leach into the sample [20]. It is not possible to avoid the influence of blood collection tubes and anticoagulants and other materials used for sample collection and storage on molecular profiles [21]. As metabolomics is a novel and developing technique, and no clear recommendations about preanalytical factors and quality control are available yet; therefore, we strongly recommend that the same collection and storage tubes should be used to collect samples for the whole study, as it was established in MUB Biobank.

## 4. Discussion

Biobank functions as a comprehensive infrastructure consisting of interdisciplinary teams responsible for specific tasks based on detailed SOPs, including patient qualification, personal and clinical data collection, and collection, storage, and quality control of biological material. During the biobanking process, each team must report on its activities and is a subject of routine control for compliance with procedures. Biobanking for challenging omics analyses requires a strict following of the scheme since the quality of samples undoubtedly strongly affects the final results [22]. Biobanks play a crucial role as a partner not only for scientists conducting their own narrowly focused projects but also for large enterprises performing research on a global scale. Certainty of high-quality collected material opens up opportunities to conduct large-scale investigations in every field of medicine, thus leading to its development. Completing data in harmonized databases allows for cooperation between particular biobanks for access to biospecimens and related data for cross-biobanking research [23]. In some cases, e.g., rare cancers, biobanks seem to constitute the only option for collecting samples for relevant studies [24].

The amount of molecular information obtained from various biospecimens depends on handling and processing the samples. Well-prepared and deposited high-quality samples allow for obtaining reliable, corresponding data for target validation and drug discovery. Analyzing this data across multiple sets can answer important biological questions about effective biomarker identification in any clinical stage of disease, also related to progression. Moreover, this information is essential to identifying disease subtypes and understanding which patients will likely respond to the candidate medications in the treatment algorithm. High-quality biobanking can lead to better results of an investigation using the multi-omics approach, effective and better-targeted clinical trials, decreased discovery costs, expanded drug development, and final effect-improved patient outcomes.

In several experiments, the influence of warm and cold ischemia time on protein and gene expression have been investigated [25,26,27]. Effects are complex since both warm and cold ischemia trigger a cascade of harmful effects and responses to hypoxia and stress, all of which affect the quality of biospecimen and subsequently of any analytical approach [26,28]. Comparing molecular changes of cold ischemia at various time points revealed that 15 min after surgery, a certain percentage of detectable genes and proteins (with reports ranging from 1% to 15%) and 30 min after surgery, up to 20% of all detectable molecules showed moderate to significant changes from baseline values [25,26,28]. Variability in tissue response is not only confounded by tissue, patient, and population heterogeneity [26] but also by the complexity of phosphorylation cascades of different proteins, hypoxia-induced responses, and posttranslational modification. The complexity of all these factors consequently results in a fluctuation of expression levels of phosphorylated proteins within the first 20 to 30 min of cold ischemic time [26,29], followed by dephosphorylation processes within 1 to 2 h of delay to fixation, leading to the significant decrease in the majority of phosphoepitopes [12,13]. Data from studying the effect of warm ischemia on the molecular composition of postsurgical tissues revealed that the severity of posttranslational modification and stress response increase with prolonged ischemic time [16]. Several vital biomarkers and therapeutic targets, such as mTOR, ERK1/2, AKT, and MEK, are upregulated within 10 min of warm ischemic time, while their expression levels subsequently decrease again [13,20]. An improved description of ischemia-induced changes may ultimately provide a better understanding of how clinically relevant markers must be interpreted and how this will affect patient stratification, staging, prognosis, and selection of appropriate targeted treatment.

Biofluids are characterized by low levels of RNA, high levels of inhibitors, and sensitivity to many preanalytical variables. Therefore, it is essential to provide specialized and optimized protocols for QC procedures. Fluorescence-based quantification assays that enable precise quantification down to as little as 1 pg/μL [30] are often preferred for challenging liquid samples. The fluorometric measurements falsely increase RNA amounts, probably due to the high concentration of contaminants (proteins, lipids) or the absence of fluorescent dye specificity, which should be intercalated or associated only with RNA. A reliable assessment of RNA concentration cannot be performed in the case of fluid samples to monitor yield and ensure consistent RNA input across all samples within the same experiment. For this reason, we used standardized input amounts both for the same volume of fluid in each RNA isolation and the same volume of extracted RNA for all samples rather than RNA quantity.

Biospecimens used for diagnostic procedures, clinical testing, or biomarker identification must be collected under strictly regulated conditions to minimize the effect of preanalytical biases on study results [31]. Therefore, professionally run biobanks with standardized procedures must obtain reliable data combined with samples collected during unified processes. As the QC plays a crucial role in biobanking specially dedicated to high-throughput studies, the integration of comprehensive QC procedures specifically designed for challenging omics analyses presented in the following paper facilitates large-scale investigations across various fields of medicine, leading to advancements in research, reduced discovery costs, expanded drug development, and improved patient outcomes.

Cooperation between biobanks, researchers, and industry is fundamental to accelerating research and integrating innovative translational activities. Nevertheless, the establishment and management of biobanks require large workloads combined with the preparation of multiple bases: ethical, legal, scientific, and clinical. Furthermore, building a committed team with well-qualified personnel aware of the whole set of procedures presents a challenge. Despite these obstacles, we need to facilitate the procedures for supporting the internationalization of access to biobank resources, since the biospecimens and data obtained in controlled and standardized conditions should be adopted for research on novel approaches to cancer prevention, early diagnosis, and personalized treatment. To facilitate access to detailed QC workflows, datasets, and practices supporting omics research, we have made efforts to ensure their availability on the MUB Biobank website in the Data Repository [32]. This initiative is a component of the Internationalization Project aimed at providing access to MUB Biobank, thereby enabling the wider scientific community to utilize the tools and methodologies developed in our study.

To further enhance the accessibility and visibility of MUB Biobank resources, which became a member of the Biobanking and Biomolecular Resources Research Infrastructure, European Research Infrastructure (BBMRI-ERIC) Consortium, in 2020, the data about collections and datasets from MUB Biobank will soon be included in the BBMRI-ERIC Directory. The BBMRI-ERIC Directory serves as a valuable platform for researchers worldwide, providing them with the means to discover and access biospecimens and related data from various biobanks [33]. By adding our collections to the BBRMI-ERIC Directory, we aim to foster collaboration and enable researchers to leverage our resources for diverse scientific investigations.

## 5. Conclusions

In summary, our study addressed the challenges posed by novel molecular techniques in biobanking for high-throughput translational research. By using biospecimens and data collected under highly standardized conditions, we developed specific quality control workflows for serum/plasma and tissue samples. These workflows, tailored to the needs and capabilities of different biobanking entities, encompass various steps, such as hemolysis monitoring, RNA extraction control, cDNA library quality assessment, and NGS raw data verification. By implementing these molecular-based quality control methods, biobanks can ensure the provision of biospecimens with confirmed omics-grade quality for advanced research applications. The workflows we proposed offer a reliable process for qualifying specimens prior to conducting multi-omics assays, demonstrating high sensitivity and specificity. This contribution to the field of biobanking promotes standardized practices, enhances research reproducibility, and fosters reliable translational research outcomes. The novelty of the presented workflows lies in their integration and comprehensive nature, providing researchers with a valuable tool for ensuring the quality of biospecimens in high-throughput research where existing guidelines may be limited or fragmented.

## Figures and Tables

**Table 1:** The nucleic acid concentration and integrity values in fresh-frozen tissue samples evaluated in the process of developing QC workflow.

	Total Number of Analyses	Concentration of Nucleic Acids (Qubit Fluorometer)		RIN	PreSeq QC Assay		Kappa Quantification	Success of NGS	
NGS_Lung_DNA	487	DNA	153 ± 38.7	none	Ct > 20 *n* = 474 (97.3%)	Qualified = 474	Pass = 473	Pass	473
			>100 ng/µL *n* = 478 (98.2%)						
			<100 ng/µL *n* = 9 (1.8%)		Ct > 20 *n* = 13 (2.7%)	Disqualified = 13	Failed = 1	Failed	14
NGS_Lung_RNA	432	RNA	63.2 ± 7.18	7–10 *n* = 428 (99.07%)	Ct > 10 *n* = 428 (99.07%)	Qualified = 428	Pass = 426	Pass	426
			>50 ng/µL *n* = 428 (99,07%)						
			<50 ng/µL *n* = 4 (0.93%)	<7 *n* = 4 (0.93%)	Ct < 10 *n* = 4 (0.93%)	Disqualified = 4	Failed = 2	Failed	6
NGS_BRCA1/2	193	DNA	326.4 ± 93,5	none	Ct >20 *n* = 193 (100%)	Qualified = 193	Pass = 193	Pass	193
			>100 ng/µL *n* = 193 (100%)		Ct < 20 *n* = 0 (0%)	Disqualified = 0	Failed = 0	Failed	0

## Data Availability

The data presented in this study are available upon request from the corresponding author. The data are not publicly available due to restrictions imposed by ethical considerations and privacy regulations to ensure the confidentiality of patient information and protect individual privacy. Requests for access to the data can be made to the corresponding author, who will consider the ethical and legal implications before granting access to the data.
